# Latent profile analysis of post-traumatic growth and psychological resilience among patients with mental illness during the rehabilitation period: associations with social support

**DOI:** 10.3389/fpsyt.2025.1738438

**Published:** 2026-01-12

**Authors:** Zifei Yang, Jianing Gu, Xiuyu Yao, Lina Wang, Jing Shao

**Affiliations:** 1 School of Nursing, Chinese Academy of Medical Sciences & Peking Union Medical College, Beijing, China; 2 Nursing Department, Beijing Huilongguan Hospital, Beijing, China; 3 Nursing Department, Hebei Mental Health Center, Baoding, Hebei, China

**Keywords:** latent profile analysis, patients with mental illness, post-traumatic growth, psychological resilience, social support

## Abstract

**Background:**

The rising number of people affected by mental health disorders contributes to a significant disease burden and presents a major public health challenge worldwide. The purpose of the present study was to explore profiles of post-traumatic growth (PTG) and psychological resilience in rehabilitation patients with mental health disorders, and to analyze the effects of social support reported by these individuals.

**Methods:**

A convenience sampling method was employed to recruit 273 patients (*M*_age_ = 37.57, SD = 12.82) hospitalized in a psychiatric specialty hospital, from August to October 2024. Participants provided data through self-report instruments, including a general information questionnaire, the Post-traumatic Growth Inventory (PTGI), the Connor-Davidson Resilience Scale (CD-RISC), and Multidimensional Scale of Perceived Social Support (MSPSS), with all items from each scale administered. Latent profile analysis was conducted to explore the potential categorization of PTG and psychological resilience; multifactorial logistic regression was used to analyze its influencing factors; and ANOVA was used to compare differences in social support level reported by patients with different categories of PTG and psychological resilience.

**Results:**

Measures of CD-RISC and PTGI displayed substantial heterogeneity, and were therefore divided into three groups: severe turmoil (13.3% of participants), fluctuating adaptation (53.6%), and integrated adaptation (33.1%). Regression analysis showed that age and social support were factors that significantly influenced classification (*P* < 0.05), and comparison of social support scores among patients in the three groups showed significant differences (*P* < 0.05).

**Conclusion:**

Heterogeneity existed in the PTG and psychological resilience of patients during the rehabilitation period. Compared with other subgroups, patients in the integrated adaptation group exhibited elevated social support resources. Healthcare professionals should utilize these groups when assessing PTG and resilience. This will allow for personalized clinical interventions based on these profiles that may help ameliorate psychological distress, increase perceived social support, and enhance overall mental well-being.

## 1 Introduction

Mental health disorders are currently a pressing global public health priority. Approximately one billion people worldwide experience some form of mental disorder (1), representing 13% of the global disease burden and the fifth highest component of the Disability Adjusted Life Years (DALYs) burden hierarchy (2). A joint World Bank-World Health Organization (WHO) report (3) estimated that economic losses due to mental health disorders in low- and middle-income countries could reach 3.5% of global GDP by 2030. Data from the China Mental Health Survey (CMHS, 2013-2015) revealed a lifetime prevalence rate of mental disorders at 16.6% among Chinese adults, indicating both the high prevalence of these conditions and their substantial disease burden (4). Mental health disorders not only result in impaired social functioning and increased costs of family care, but can also cause strain on public health resources.

Through systematic treatment, a substantial proportion of patients report improvement in symptoms, and thus enter the rehabilitation phase, which can sometimes be characterized by residual impairments in psychosocial functioning, despite symptom improvement. During this critical rehabilitation phase, many patients struggle with trauma adaptation deficits, manifesting as compromised post-traumatic growth (PTG) and diminished psychological resilience (5).

PTG, which is conceptualized through the refined functional-descriptive conceptual framework (6), entails positive psychological transformation that is driven by cognitive restructuring following trauma, wherein individuals reconstruct meaning through narrative processing and schema revision. This process manifests in three domains: renewed self-perception, relational connectedness, and existential meaning reconstruction (6). Neurobiological studies (7) have shown that PTG involves prefrontal cortex-mediated reappraisal mechanisms. Additionally, defined as a dynamic stress-adaptation capacity, psychological resilience enables individuals to utilize biological, psychological, and social resources to maintain homeostasis in response to adversity (8). Richardson’s (9) metatheoretical framework posits resilience as an emergent outcome of successful resource integration across these systems, which facilitates functional recovery. Understandably, PTG and resilience share underpinnings in cognitive-emotional integration. PTG necessitates resilience to sustain meaning-making processes during cognitive reappraisal (10), while resilience potentiates PTG through mitigation of trauma-induced allostatic load (11). Critically, meta-analytic evidence suggests that patients with mental health disorders typically exhibit significantly lower PTG (12) and reduced resilience (13) when compared to healthy controls. This dual deficit exacerbates psychological distress, prolongs functional disability, and elevates relapse risk (5).

Social support is crucial for trauma adaptation. It refers to the help people receive from their social networks, whether emotional, informational, or practical (14). According to Cohen and Wills’ Stress-Buffering Theory (15), social support can soften the negative impact of stress. It helps people cope better and reframe their experiences positively. In mental health recovery, strong social support builds resilience. It gives individuals a sense of belonging and boosts their confidence (16). It also encourages PTG by allowing them to share emotions and find new meaning (17). Many studies confirm that more social support is linked to higher resilience and greater PTG in people facing trauma or illness (18). Yet, we still know little about how social support works together with PTG and resilience in mentally ill patients during rehabilitation (16). This gap calls for more focused research. As Cohen’s (15) theory suggests, social support helps through practical aid, emotional comfort, and useful advice. On the other hand, lacking support is tied to poorer trauma responses. For example, it can increase amygdala reactivity to threats (19) and weaken prefrontal regulation (20).

Empirical evidence (21) has shown that social support enhances capacity of patients to cope with trauma and bolsters psychological resilience. However, despite social buffering theory indicating that social factors can affect individuals’ psychological status, few studies have explored the impact on PTG and resilience in patients with mental illness during the rehabilitation period (22). Current research on PTG, as well as psychological resilience, has primarily focused on status quo investigations (5, 8), with researchers consistently finding that psychiatric populations exhibit compromised development of PTG and diminished psychological resilience capacities, both of which influence the speed of recovery and the ability of patients to effectively reintegrate into society (23). Furthermore, research examining the correlations among PTG, psychological resilience, and social factors remain limited (22). This evidence gap necessitates systematic exploration in mental health patients of the dynamic interplay between psychological homeostasis and social support in the face of adversity.

Traditional statistical approaches like regression analysis have been valuable for examining PTG and psychological resilience as independent variables. However, these methods cannot adequately capture how these constructs interact within individuals—a critical consideration when studying trauma adaptation in psychiatric rehabilitation patients, where recovery patterns vary substantially between individuals (24). We chose latent profile analysis (LPA) for this study because it addresses three key limitations of conventional approaches: First, unlike variable-centered methods that examine factors in isolation, LPA identifies subgroups of individuals who share similar patterns across multiple dimensions (25). This is particularly important for understanding how PTG and resilience co-occur in different patient populations. Second, LPA accounts for the nuanced reality of mental health recovery, where patients rarely fit into absolute categories but instead exhibit varying degrees of adaptive functioning (26). Third, the empirically derived profiles generated by LPA have immediate clinical relevance, allowing healthcare providers to tailor interventions to specific patient needs (e.g., intensified social support for patients in the “severe turmoil” profile versus maintenance strategies for those in “integrated adaptation”).

Guided by Richardson’s dynamic model of resilience (9), we proposed three specific hypotheses: First, that patients would cluster into distinct profiles based on their PTG-resilience configurations. Second, that these profiles would demonstrate different levels of perceived social support. Third, that profile membership would be associated with differential rehabilitation outcomes. Our results supported all three hypotheses, identifying three meaningful subgroups that differed significantly in both their support needs and clinical trajectories.

## 2 Methods

### 2.1 Study design and participants

The current investigation used convenience sampling to select 273 psychiatric patients hospitalized at a mental health center in Hebei Province, China from August to October 2024. The sample size was determined based on prior studies and statistical considerations. Existing research on PTG and psychological resilience (27) in psychiatric patients has commonly included 8–10 key influencing factors. According to the guideline that sample size should be 10–15 times the number of predictors in regression analysis, an initial target of 80–150 participants was derived. More critically for the primary analysis, LPA necessitates adequate sample sizes for reliable subgroup detection and statistical power. Simulation studies (28) recommend at least 200 participants to ensure profile stability, classification accuracy, and adequate power (>0.80) to correctly reject false models. Our final sample of 273 well exceeds this threshold, providing sufficient statistical power for the LPA. Convenience sampling is based on the principles of accessibility and time-efficiency, prioritizing the inpatient group with optimal spatial and temporal accessibility as the sample source. Although this method involves the risk of selection bias, it can effectively match the resource constraints of psychiatric clinical research and the need for immediate data acquisition.

### 2.2 Sampling: inclusion and exclusion criteria

Inclusion Criteria: (1) meets diagnostic criteria of psychiatric disorders, including schizophrenia, bipolar disorder, depression, or mania by psychiatric specialists; (2) being in the recovery phase, characterized by clinical stability, improved symptoms, and recovered self-awareness as determined by the attending physician; (3) hospitalization time ≥ 2 weeks, ensuring patients had moved beyond the initial acute stabilization phase and had sufficient exposure to the rehabilitation environment to begin the process of psychological adaptation; and (4) voluntary participation. This study excluded individuals who presented with mental health disorders in combination with other acute or severe somatic diseases, as well as those who exhibited cognitive impairment and could not communicate effectively.

### 2.3 Research tools

#### 2.3.1 General information questionnaire

A general information questionnaire was compiled by the researchers following a review of the relevant literature. Sociodemographic data were collected through structured instruments, encompassing age, gender, marital status, location of residence, education level, occupation, monthly income, medical expenses, and method of payment.

#### 2.3.2 Post-traumatic growth inventory

The PTGI was developed by Tedeschi and Calhoun (29) to quantitatively assess, via self-report, positive psychological shifts in individuals following traumatic events. The psychometrically robust PTGI-21 instrument categorizes 21 items into 5 theoretically grounded dimensions: novel opportunity realization; relational intensification; endurance fortification; existential reappraisal; and transcendental awareness. This psychometric instrument employs a 6-point Likert scale (0 = *no experienced change*; 5 = *profound change*). The theoretical scoring spectrum spans 0–105 points, with elevated composite scores indicating greater PTG. Psychometric analyses have demonstrated robust internal consistency (Cronbach’s α = 0.90 for total scale), confirming the measure’s empirical reliability and construct validity. Wang et al. (30) introduced the PTGI into the Chinese context by translating, culturally adapting, and validating the instrument for use among accidental trauma survivors in mainland China. The reliability of the Chinese version of the PTGI was robust, with a Cronbach’s α coefficient of 0.874 for the total scale. Notably, the revised PTGI has gained widespread recognition and application across China. In the current sample, the Chinese PTGI exhibited good internal consistency, with Cronbach’s α of 0.979 for the total scale, and 0.823-0.947 for the five dimensions.

#### 2.3.3 Connor-Davidson resilience scale

The standard CD-RISC (31) consists of 25 self-reported items, divided into five empirically derived factors based on exploratory factor analysis: personal competence and tenacity; trust in instincts and tolerance of negative affect; positive acceptance of change and secure relationships; and control and spiritual influences. The scale measures core abilities to maintain psychological homeostasis under adversity, focusing on stress coping efficacy and resource mobilization. It uses a 5-point Likert scale (0 = *not true at all*; 4 = *true nearly all the time*), with total scores ranging from 0 to 100, with higher scores indicative of greater resilience. The CD-RISC total score is widely utilized as a composite, unidimensional measure of overall resilience in diverse populations, including Chinese samples, and its validity for this purpose is well-established (32). Consistent with this practice and the objective of our latent profile analysis—to categorize participants based on their global levels of post-traumatic growth and resilience—we used the total scores of the CD-RISC and PTGI as continuous indicators. In our sample, the CD-RISC demonstrated excellent internal consistency, with a Cronbach’s α of 0.963 for the total and 0.694-0.925 for the five factors.

#### 2.3.4 Multidimensional scale of perceived social support

The MSPSS (33) is a psychometrically validated self-report instrument quantifying subjective social support appraisal. It comprises 12 items partitioned into 3 factors: family support, friend support, and significant other support. These subscales operationalize perceptions of emotional and instrumental support adequacy within distinct social networks. Respondents rate items on a 7-point Likert scale (1 = *strongly disagree*; 7 = *strongly agree*), yielding aggregate scores ranging from 12–84. The psychometrically adapted Chinese version (34) exhibits robust reliability (α = 0.89) and criterion validity, establishing its utility in stress-coping and social resource allocation research paradigms. In the current study, the scale demonstrated excellent internal consistency, with a Cronbach’s α of 0.946 for the total score and ranging from 0.864 to 0.88 for the subscales.

### 2.4 Data collection and quality control

To ensure procedural standardization and minimize social desirability bias, all staff received uniform training to deliver neutral instructions. Additionally, participants were guaranteed anonymity and their voluntary, penalty-free right to withdraw was emphasized to encourage candid responses. During data collection, trained investigators and primary nurses administered questionnaires in standardized 15-minute sessions, based on pre-test results showing an average completion time of 15.6 ± 3.2 minutes. If participants were unable to finish within 15 minutes, they were offered the option to pause and resume after a brief rest period. The study involved 279 patients in the recovery phase of mental illness who met strict inclusion criteria: demonstrated clinical stability (evidenced by reduced treatment frequency), preserved cognitive function (MMSE score ≥24), and capacity to provide informed consent. The informed consent process involved three key steps: (1) a verbal explanation using simplified, large-print educational materials, (2) a comprehension assessment where participants were asked to explain the study requirements in their own words, and (3) formal written consent. Throughout the process, clinical staff carefully monitored participants for any signs of fatigue, including reduced attention span or increased restlessness, and allowed multiple days to complete the survey if necessary. Structured paper questionnaires served as the primary modality for patient-reported data collection. Completed questionnaires were collected on site. A total of 279 questionnaires were distributed in this study. Six participants were excluded from the analysis due to substantial amounts of missing data in critical fields. Ultimately, 273 valid questionnaires were collected, with a valid recovery rate of 97.85%.

Patient confidentiality was strictly maintained throughout the study. All collected questionnaires were anonymized immediately upon collection by replacing personal identifiers with a unique code. The electronic database containing the study data was accessible only to the principal investigators. All paper records, including the original questionnaires and consent forms, were stored in a locked cabinet in a secure office. Any potentially identifiable information was removed from the dataset prior to analysis.

### 2.5 Data analysis

#### 2.5.1 Common method deviation test

Investigators ensured that participants completed all paper questionnaires within the same context to obtain data. However, this process may result in common method bias. Therefore, to pre-assess systematic interference of common method variance on the measurement results, this study used the Harman one-way test for statistical control.

#### 2.5.2 Confirmatory factor analysis

We conducted confirmatory factor analysis (CFA) using Mplus 8.3 to examine the structural validity of the three key scales: the Post-traumatic Growth Inventory (PTGI), Connor-Davidson Resilience Scale (CD-RISC), and Multidimensional Scale of Perceived Social Support (MSPSS). The hypothesized factor structures were tested for each scale. Model fit was evaluated using several standard indices: χ²/df ratio (values < 3 indicating good fit), comparative fit index (CFI > 0.90), Tucker-Lewis index (TLI > 0.90), root mean square error of approximation (RMSEA < 0.08), and standardized root mean square residual (SRMR < 0.08). All items were required to have factor loadings exceeding 0.50 and be statistically significant (*P* < 0.001).

#### 2.5.3 Descriptive and inferential statistical analyses

Statistical analyses were performed using SPSS 27 for descriptive statistics, inferential tests (α= 0.05), and regression analyses, while Mplus 8.3 was used exclusively for LPA. The assumption of univariate normality for the LPA indicator variables (PTG and psychological resilience total scores) was examined and supported the presence of latent profiles. For the subsequent group comparisons based on the derived profiles, normally distributed continuous data were expressed as Mean ± SD and analyzed by a one-way analysis of variance (ANOVA), while categorical variables were expressed as frequencies (%) and compared using χ^2^/Fisher’s tests. Variables demonstrating statistical significance (*P* < 0.05) in univariate analyses were subsequently entered into the multivariate logistic regression model.

#### 2.5.4 Latent profile analysis

Descriptive statistics were recorded and Pearson correlations were conducted between study variables. Next, LPAs were conducted, including all items of PTGI and CD-RISC. Models were estimated with 1 to 6 levels of resolution without incorporation of any other model specifications. The fitness metrics we chose for the profile models included the Akaike information criterion (AIC), Bayesian information criterion (BIC), and sample-size adjusted Bayesian information criterion (saBIC), with smaller values indicating a better model fit. If the difference between the bootstrap likelihood ratio test and the Lo-Mendell-Rubin test was significant (*P* < 0.05), then the k-category model was considered to outperform the k-1 category. Entropy was used to measure model categorization accuracy, with values closer to 1 indicating higher categorization accuracy.

### 2.6 Validity and reliability

The research protocol was rigorously aligned with the strengthening the reporting of observational studies in epidemiology (STROBE) statement. Specifically, elements regarding the standardized reporting criteria for observational studies, prioritizing both participant rights preservation and methodological transparency.

## 3 Results

### 3.1 Common method deviation test

Results showed 5 common factors with eigenvalues > 1 were extracted by Exploratory Factor Analysis (EFA), and the explanatory rate of the first common factor was 18.2% (the critical value was less than 40.0%). Therefore, the correlations between the variables in this study were largely unaffected by common method bias.

### 3.2 Confirmatory factor analysis results

The confirmatory factor analysis supported the structural validity of all three scales in our sample. The MSPSS three-factor model showed excellent fit (χ²/df = 2.45, CFI = 0.95, TLI = 0.94, RMSEA = 0.057, SRMR = 0.039). The PTGI five-factor model demonstrated good fit (χ²/df = 2.86, CFI = 0.92, TLI = 0.91, RMSEA = 0.063, SRMR = 0.045), as did the CD-RISC five-factor model (χ²/df = 3.12, CFI = 0.91, TLI = 0.90, RMSEA = 0.068, SRMR = 0.051). All items loaded significantly on their intended factors (*P* < 0.001), with standardized loadings ranging from 0.55 to 0.90. Although the CFA for the CD-RISC produced a technical warning (possibly due to high correlations among factors), the overall model fit was acceptable and all factor loadings were strong, supporting the validity of the scales in our analysis.

### 3.3 Participants and general demographic characteristics

Complete data of 273 cases were analyzed in this study, with a mean age of (37.57 ± 12.82) years old. The general information is displayed in **Table 1**.

**Table 1:** General information on patients (n=273).

Profile		Frequency	Proportion (%)
Genders	Male	121	44.3
	Female	152	55.7
Marital Status	Spousal	148	54.2
	Spouse-less	125	45.8
Current Address	Municipalities	85	31.1
	Countryside	168	61.5
	City and Countryside Integration	20	7.3
Education Level	Primary School and Below	38	13.9
	Junior High School	69	25.3
	Technical Secondary School/High School/Technical Secondary School	101	40
	University (Junior College or Undergraduate) and Above	65	23.8
Employment Status	Having A Stable Job	75	27.5
	Having No Fixed Job	182	66.7
	Retirement	16	5.9
Monthly Income (RMB)	<2000	65	22.7
	2000~4999	125	45.8
	4000~6999	62	22.7
	≥7000	24	8.8
Payment Methods	Medical Insurance	156	57.1
	New Agricultural Cooperative Society (NACS)	100	36.6
	Self-financed	15	5.5
	At Public Expense	2	0.7
Residency	Living Alone	42	15.4
	Living With Spouse	121	44.3
	Living With Parents/Children	110	40.3

### 3.4 Results of LPA

Pearson correlation analysis revealed significant positive correlations between the total scores of PTG and psychological resilience (r = 0.170, *p* = 0.005), indicating that higher levels of PTG were associated with higher levels of resilience. The analytical results of LPA are displayed in **Table 2**. Model 3 was selected as the optimal solution through a multi-criteria decision framework. Although the 4-to-6-class models demonstrated marginally superior statistical fit (log-likelihood closest to zero, lowest AIC/BIC/saBIC), Model 3 was favored due to its high entropy (0.98), significant improvement over Model 2 (LMR & BLRT, *P* < 0.01), and superior parsimony and substantive interpretability. The higher-class models yielded at least one subgroup with a very small size (<8%), which was deemed less stable and theoretically meaningful for our sample.

**Table 2:** Joint Potential profile model fit for PTG and psychological resilience in people with mental illnesses (n=273).

Profile	Likelihood	AIC	BIC	saBIC	Entropy	LMR(p)	BLRT(p)	Proportion
1	-19668.86	39521.73	39853.80	39562.09	1.00			1
2	-18187.05	36652.10	37153.82	36713.08	0.99	<0.001	0.01	0.643/0.357
3	-17101.30	34574.61	35245.97	34656.21	0.98	<0.001	0.01	0.133/0.536/0.331
4	-16513.35	33492.70	34333.71	33594.93	0.99	<0.001	0.01	0.121/0.528/0.274/0.077
5	-15715.49	31990.99	33001.64	32113.83	0.99	<0.001	0.01	0.128/0.165/0.463/0.186/0.059
6	-15470.19	31594.38	32774.67	31737.84	0.97	<0.001	0.01	0.120/0.150/0.291/0.149/0.070/0.220

AIC is the Aicheck information criterion; BIC is the Bayesian information criterion; saBIC is the scaling-adjusted BIC; Entropy is the information entropy; LMR is the Lomondale-Reuben-corrected likelihood ratio; BLRT is the Bootstrap-based likelihood ratio test.

**Figure 1:**
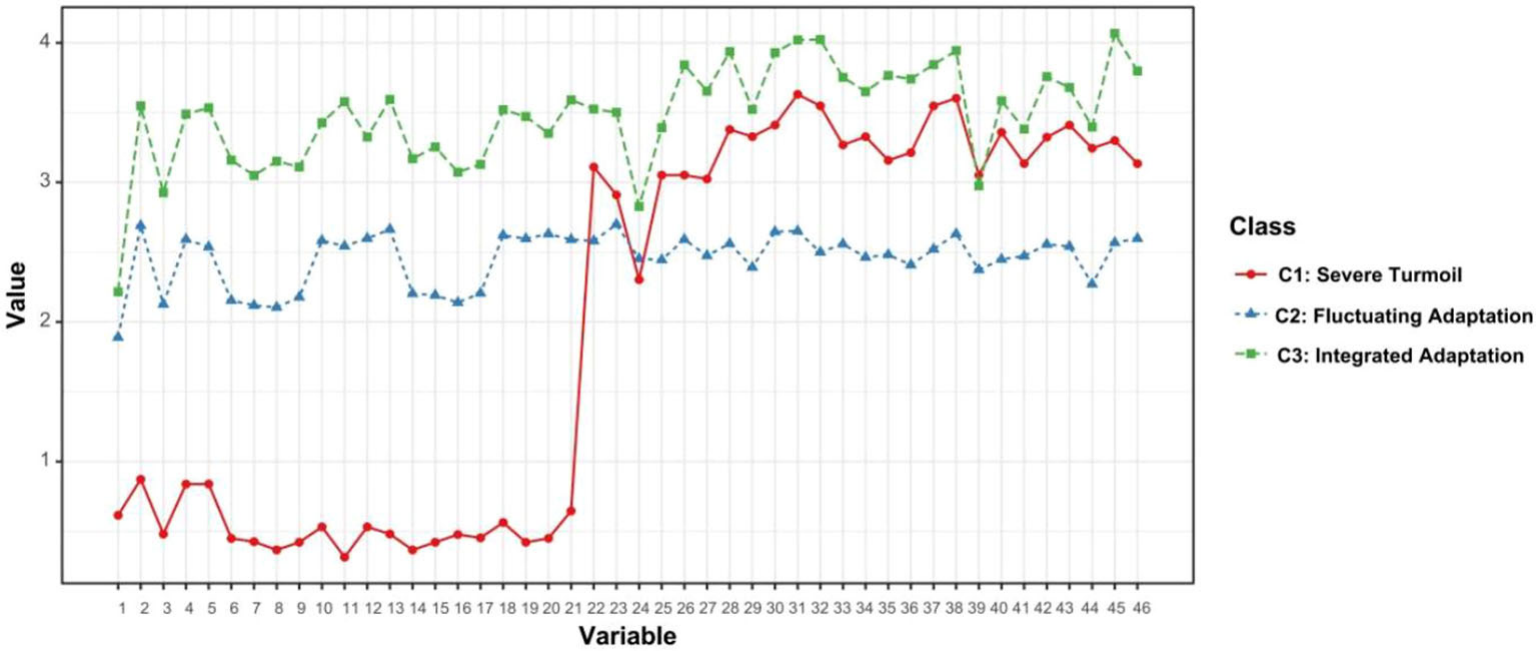
Joint potential profiles of PTG and psychological resilience in people with mental illnesses.

Potential profiles were plotted based on the categorization results (**Figure 1**). Three categories were named based on the mean values of the dimension scores. (1) C1: lowest score (PTGI = 10.83 ± 10.87, CD-RISC = 80.97 ± 23.81), and negatively predictive subgroup for social support (β = −0.109). Therefore, it was named classified as the severe turmoil group (2) C2: medium core variables (PTGI = 49.95 ± 10.29, CD-RISC = 62.90 ± 11.51), social support was weakly correlated with PTG (r = 0.31), and it was named as fluctuating adaptation group. (3) C3: The highest scores of PTG and psychological resilience (PTGI = 68.68 ± 17.14, CD-RISC = 91.58 ± 14.96), social support was significantly higher than the other groups (MSPSS = 65.26 ± 12.77, *P* < 0.001), and named as the integrated adaptation group.

The percentages of the three categories were 13.3% (n = 36), 53.6% (n = 147), and 33.1% (n = 90).

### 3.5 Comparison of social support among the three latent profiles

Comparison of the dimensions of social support and total scores of the three groups of patients with mental disorders showed statistically significant differences (**Table 3**). *Post-hoc* comparisons using Tukey’s HSD test revealed that for all social support variables, the Fluctuating Adaptation group (C2) scored significantly lower than both the Severe Turmoil group (C1) and the Integrated Adaptation group (C3). However, there were no significant differences in social support scores between the Severe Turmoil (C1) and Integrated Adaptation (C3) groups.

**Table 3:** Comparison of social support in joint potential profiles of PTG and psychological resilience in patients with mental disorders.

Class	Family support	Friends support	Other cooperation	Social support
Class1	21.17 ± 4.09	20.25 ± 4.54	21.19 ± 3.98	62.61 ± 11.44
Class2	17.97 ± 4.06	17.12 ± 3.94	17.32 ± 3.64	52.40 ± 10.43
Class3	22.20 ± 4.54	21.44 ± 4.61	21.61 ± 4.54	65.26 ± 12.77
F	30.248	30.871	37.040	39.018
*P*	<0.001	<0.001	<0.001	<0.001
*Post-hoc* Comparisons (Tukey’s HSD)	C2 < C1, C3; C1 = C3	C2 < C1, C3; C1 = C3	C2 < C1, C3; C1 = C3	C2 < C1, C3; C1 = C3

C1 = Severe Turmoil Group; C2 = Fluctuating Adaptation Group; C3 = Integrated Adaptation Group. The symbol “<“ indicates a statistically significant difference (*P* < 0.05). The symbol “=“ indicates no significant difference.

### 3.6 Univariate analysis of joint potential categories

The differences among the three categories of psychological distress trajectories of patients with mental illness were statistically significant (*P* < 0.05) in terms of gender, literacy, occupation, family income, and perceived social support (**Table 4**).

**Table 4:** Univariate analysis of joint potential categories of PTG and psychological resilience for people with mental illnesses.

Variables	Potential categories			χ2/F	*P*
	Class 1(*n* = 36)	Class 2(*n* = 147)	Class 3(*n* = 90)		
Genders				18.118^1)^	<0.001
Male	19 (52.78)	48 (32.65)	54 (60.00)		
Female	17 (47.22)	99 (67.35)	36 (40.00)		
Education level				15.348^1)^	0.018
Primary school and below	5 (13.89)	25 (17.01)	8 (8.89)		
Junior high school	12 (33.33)	39 (26.53)	18 (20.00)		
Technical secondary school/High school/Technical secondary school	11 (30.56)	59 (40.14)	31 (34.44)		
University (junior college or undergraduate) and above	8 (22.22)	24 (16.33)	33 (36.67)		
Employment status				10.782^1)^	0.029
Having a stable job	12 (33.33)	29 (19.73)	34 (37.78)		
Having no fixed job	23 (63.89)	109 (74.15)	50 (55.56)		
Retirement	1 (2.78)	9 (6.12)	6 (6.67)		
Monthly income (RMB)				19.276^1)^	0.004
<2000	9 (25.00)	32 (21.77)	21 (23.33)		
2000~4999	10 (27.78)	78 (53.06)	37 (41.11)		
4000~6999	8 (22.22)	30 (20.41)	24 (26.67)		
≥7000	9 (25.00)	7 (4.76)	8 (8.89)		
MSPSS	62.61 ± 11.45	52.40 ± 10.43	65.26 ± 12.77	39.018^2)^	<0.001

1) χ^2^ value; 2) F value.

### 3.7 Multivariate logistic regression of joint trajectory profiles

With psychological distress in patients with mental disorders as the dependent variable (assigning values of 1, 2, and 3 to the severe turmoil group, the fluctuating adaptation group, and the integrated adaptation group, respectively, with the integrated adaptation group as the reference and the other three groups as the outcome variables), and variables with statistically significant differences in the one-way ANOVA as the independent variables, the independent variables were assigned as follows: gender (male = 0, female = 1); education level (elementary school and below = 1, and junior high school = 2, high school/high school/secondary school = 3, university [college or bachelor’s degree] and above = 4); occupation (regular job = 1, no regular job = 2, retired = 3); and per capita monthly household income (< 2000 = 1, 2000 –4999 = 2, 4000–6999 = 3, ≥ 7000 = 4). The total score of perceived social support was treated as a continuous variable in the multivariate logistic regression analysis. The results showed that age and perceived social support were influential factors in the fluctuating adaptation group (*P* < 0.05), using the integrated adaptation group as a reference (**Table 5**).

**Table 5:** Multivariate logistic regression analysis of the combination of traumatic growth and psychological resilience in patients with mental illness.

Dependent variable	Independent variable	β	SE	Wald2	*P*	OR	95%CI
C1 vs.C3	Intercept	-11.984	4929.049	<0.001	0.998	—	—
C2 vs.C3	Intercept	-17.215	2.865	36.108	<0.001	—	—
	Age	0.051	0.019	7.107	0.008	1.053	1.014~1.093
	PSSS	-0.109	0.017	40.137	<0.001	0.896	0.867~0.927

## 4 Discussion

This study identified three distinct subgroups of individuals with mental illness in the recovery period characterized by varying levels of PTG and psychological resilience. Crucially, perceived social support emerged as a significant predictor of subgroup membership, with the integrated adaptation group exhibiting significantly higher support compared to the other subgroups. Overall, these findings underscore the heterogeneity in psychological adaptation following trauma and highlight the critical role of social support as a modifiable factor for targeted interventions to enhance resilience and reduce psychological distress in this vulnerable population.

### 4.1 Potential adaptation profiles in mental illness: dual deficits of PTG and psychological resilience

The present study demonstrated that individuals diagnosed with mental illness exhibit significantly lower levels of PTG and psychological resilience in comparison to normative data from the general population. This discrepancy suggests the presence of systemic barriers to trauma adaptation experienced by individuals with mental illness. The observed disparity may arise from multidimensional interactions between psychopathological mechanisms and adaptive psychological processes. Specifically, core symptoms of mental disorders, such as cognitive dysfunction and emotional dysregulation directly compromise the capacity of patients to engage in active cognitive reappraisal of traumatic experiences (35, 36). This impairment hinders the psychological reconstruction necessary for fostering PTG (37), thereby restricting perceived gains in other domains, such as “exploration of new possibilities” and “redefinition of life purpose.”

Furthermore, deficient social support serves to compound the aforementioned discrepancy. The present findings indicate that the PTGI and CD-RISC scores of the low social support group were significantly lower than those of the high support group. This observation suggests that patients may encounter challenges in mobilizing external resources that can help to mitigate traumatic stress due to the absence of robust social networks.

PTG and psychological resilience hold dual importance to the recovery of patients with mental disorders. PTG provides individuals with intrinsic motivation for psychological adaptation and long-term recovery through facilitation of the reconstruction of positive meanings from trauma (38); whereas psychological resilience helps individuals to rapidly restore functioning and reduce the risk of relapse through maintenance of psychological homeostasis in the face of adversity (39). The integrated adaptation group demonstrated optimal recovery outcomes in this study, aligning with theoretical frameworks positing that robust social support systems strengthen trauma recovery through multi-resource pathways. Specifically, emotional and instrumental scaffolding collectively enhance adaptive capacities through reinforcement of cognitive reappraisal and stress buffering. Consequently, deploying targeted interventions for socially isolated patients, such as community-based partnerships and skills-integrated Cognitive-Behavioral Therapy (CBT) groups may optimize PTG and resilience trajectories, thereby catalyzing functional reintegration and sustained remission.

### 4.2 Latent profile classification analysis of trauma adaptation deficits in psychiatric patients

The LPA revealed three distinct subgroups based on psychological functioning during trauma adaptation in people recovering from mental illnesses. Deeper analysis of our findings revealed that the severe turmoil group showed greater vulnerability on all dimensions, displaying lower scores on PTG and psychological resilience, exhibiting weaker psychological functioning in response to frustration, and reporting lower levels of perceived social support compared to the integrated adaptation group. In contrast, patients in the integrated adaptation group had significantly stronger psychological functioning in the face of frustration and reported the highest levels of perceived social support. The fluctuating adaptation group, which had the highest proportion of people, fell between these two subgroups and was intermediate on psychological functioning, but had the lowest reported level of perceived social support. We propose that this finding is uniquely illuminated by the Chinese cultural context. In China’s collectivist society, mental illness stigma is profoundly tied to “losing face”, which can trigger social withdrawal and reluctance to seek help. The internalized stigma scores of Chinese patients are higher than those reported in the West (40), directly hindering the mobilization of social resources needed for adaptive psychological resilience. Individuals in the fluctuating adaptation group, characterized by psychological ambivalence, may be particularly susceptible to this dynamic. They might lack the robust resilience of the integrated group to counter stigma, yet be more cognizant of their social needs than the severe turmoil group, thereby perceiving their isolation more acutely. This cultural mechanism also helps explain the notably high proportion of this group compared to Western studies (41). Many Chinese patients are caught between the need for support and a socio-cultural environment that inhibits its expression, resulting in a large cohort stabilized in an unstable state.

These findings are consistent with previous studies using LPA (42, 43) that have emphasized the role of social support in subgroup moderation. Person-centered approaches (44) are essential for considering the heterogeneity of responses among people with mental illness, providing valid insights into subgroup-specific risk factors and intervention strategies that may be overlooked by the use of variable-oriented approaches.

### 4.3 Factors that influence PTG and psychological resilience

The findings of the present study indicate that age is a significant factor during trauma adaptation in patients with mental illness. This suggests that advancing age functions as a risk factor for the fluctuating adaptation group. This phenomenon may be associated with a decline in neuroplasticity and cognitive functioning in older patients. As aging is accompanied by a decline in prefrontal functioning and a reduction in hippocampal volume, post-traumatic cognitive reorganization may be impaired, limiting the ability of individuals to integrate positive meaning of the traumatic event (45, 46). Therefore, it is recommended that clinics direct greater attention to the adaptation of trauma in older adult patients and to the development of community support programs for older adult patients.

Furthermore, our univariate analyses revealed several significant demographic differences among the profiles. The integrated adaptation group had a higher proportion of males, individuals with university education, and those with stable employment. These findings are consistent with existing literature suggesting that higher education and employment stability provide cognitive resources and socioeconomic advantages that facilitate better trauma adaptation (47). The gender difference observed in our study contrasts with some Western findings that report higher resilience in females (48), possibly reflecting cultural variations in gender roles and coping strategies within Chinese society. The monthly income distribution also showed notable patterns, with the integrated adaptation group having the highest proportion of high-income earners. This economic advantage may provide access to better mental health resources and reduce daily stressors, thereby creating more favorable conditions for psychological adaptation (49). These demographic patterns emphasize the importance of considering socioeconomic factors when developing targeted interventions for different patient subgroups.

### 4.4 Effect of social support on PTG and psychological resilience

The findings of this study indicate that the perception of social support functions as a protective factor for PTG and psychological resilience. Specifically, patients in the integrated adaptation group exhibited a significantly higher total perceived social support score in comparison to those in the other two groups. The hypothesis that social support can mitigate the adverse effects of stress on psychological functioning has been proposed (14). This mitigation is thought to occur through the provision of emotional resources, such as empathic listening, and instrumental resources, such as financial assistance (50). In this study, perceived social support was found to have a significant positive predictive value for patients in the integrated adaptation group. This finding suggests that patients in a high-support environment are more likely to be able to buffer traumatic shocks, which promotes cognitive restructuring that occurs through the utilization of resources (51). Previous research (6) has emphasized that traumatic growth requires the reconstruction of meaning through social interaction. Therefore, it is recommended that community workers provide home visit services to strengthen instrumental support for trauma adaptation, thus promoting recovery and return to society.

Additionally, the current study revealed discrepancies in the perceived social support received by patients with varying profiles of PTG, indicating a strong correlation between the perceived social support levels of patients with mental disorders and their degree of PTG and psychological resilience. The correlations between PTG and psychological resilience, as well as between PTG and the level of social support received by patients, were both positive and significant. This relationship may be bidirectional. On one hand, social support offers emotional and instrumental resources—such as family care and economic assistance—that help buffer the impact of traumatic stress (52). This protective effect makes it easier for individuals to reframe their experiences, thereby promoting cognitive restructuring and adaptive recovery. On the other hand, individuals with high psychological resilience are typically more capable of mobilizing their social networks. They achieve this by strengthening interpersonal communication and participating in group activities (53), which in turn fosters a positive cycle where resilience and social support reinforce each other.

Notably, our analysis of social support dimensions revealed culturally specific patterns. Family support demonstrated a larger impact on PTG and psychological resilience than friend support, which contrasts with Western studies that often report equivalent effects from peer networks (54). This finding supports the Confucian emphasis on family centrality in Chinese culture (55) and suggests that family-based interventions may be particularly effective for this population. The dominance of family support also helps explain the comparable overall support levels between the severe turmoil and integrated adaptation groups. We speculate that both groups rely heavily on family networks, but critical differences likely exist in the quality and psychological efficacy of that support. The integrated group may experience more empowering familial interactions, whereas the support for the severe turmoil group might be more instrumental or custodial, failing to adequately foster psychological growth.

### 4.5 Clinical implications and future directions

Based on our profile-specific findings, we recommend differentiated intervention approaches. For the severe turmoil group, intensive family-focused interventions combined with professional psychological support are crucial. For the fluctuating adaptation group, community-based programs that enhance social connectivity and provide skills training may be most beneficial. The integrated adaptation group may benefit from maintenance strategies and peer support roles. Future research should explore the developmental trajectories of these profiles longitudinally, particularly investigating why certain demographic groups are overrepresented in specific profiles and how cultural factors moderate these relationships. The high percentage of fluctuating adapters in our Chinese sample compared to Western studies (41) warrants further cross-cultural investigation.

### 4.6 Limitations

This investigation acknowledges methodological constraints inherent to its design. First, dependence on retrospective self-disclosure instruments introduces risks of recall bias, potentially compromising measurement precision and internal validity. Additionally, geographical constraints of surveying patients in a single psychiatric specialty hospital may impede extrapolation to the full heterogeneity of individuals with psychiatric conditions. Due to the practical limitations of clinical data collection, clinical variables such as diagnostic subtypes, disease severity, treatment adherence, and disease duration were not included in this study. Their omission may affect the interpretation of profile differences and outcomes. Future research can combine the electronic medical record system to improve the collection of this part of the data. Moreover, the cross-sectional design inherently precludes longitudinal assessment of trauma adaptation trajectories. We selected ANOVAs and multivariate logistic regression for our *post-hoc* analyses, which was appropriate given the study’s exploratory goal of characterizing the identified profiles. For future research investigating directional relationships or developmental trajectories, the use of specialized methods such as the BCH (Bolek, Croon, and Hagenaars) and R3STEP procedures in Mplus—which account for the classification uncertainty inherent in LPA—would be advantageous. It is worth noting that during the CFA, we observed a technical warning related to the latent variable covariance matrix for the CD-RISC. This is not uncommon in complex models and may reflect particularly high correlations between certain resilience factors in our clinical sample. However, given the overall strong model fit, significant factor loadings, and the well-established validity of the CD-RISC in diverse populations, we are confident that this does not undermine the validity of our findings. Future studies with larger samples could further explore the factor structure of resilience in psychiatric rehabilitation populations.

Furthermore, the cross-sectional nature of our data inherently limits causal inference. While we identified significant associations between social support, PTG, and psychological resilience, the temporal precedence and directional relationships among these variables cannot be established. Future research should prioritize longitudinal designs to track the dynamic trajectories of PTG and psychological resilience throughout the rehabilitation process and beyond. This would allow for a better understanding of how these profiles evolve over time and what factors predict transitions between subgroups. While our study employed a person-centered latent profile analysis to identify heterogeneous subgroups, it did not extensively explore the variable-centered relationships, such as mediation or moderation mechanisms within each profile. Future research would benefit from integrating person-centered and variable-centered approaches, such as conducting mediation analyses within subgroups or using network analysis, to unravel the complex psychosocial mechanisms underlying different adaptation trajectories.

## 5 Conclusions

This study aimed to identify distinct profiles of PTG and psychological resilience among patients with mental illness in the rehabilitation phase and examine their associations with social support. Latent profile analysis revealed three distinct subgroups, confirming the presence of significant heterogeneity in these psychological resources. Critically, perceived social support emerged as a key factor differentiating these profiles, with the integrated adaptation group reporting significantly higher levels than the other groups. These findings emphasize that social support is integral to fostering adaptive PTG and resilience during psychiatric rehabilitation. Clinicians should prioritize assessing patients’ psychological resilience profiles and social support networks to tailor individualized interventions, ultimately enhancing psychological well-being and recovery outcomes.

## Data Availability

The raw data supporting the conclusions of this article will be made available by the authors, without undue reservation.

## Glossary

AIC: Akaike Information Criterion
ANOVA: Analysis of Variance
BIC: Bayesian Information Criterion
CBT: Cognitive-Behavioral Therapy
CD-RISC: Connor-Davidson Resilience Scale
CFA: Confirmatory Factor Analysis
CMHS: China Mental Health Survey
DALYs: Disability Adjusted Life Years
EFA: Exploratory Factor Analysis
GDP: Gross Domestic Product
LPA: Latent Profile Analysis
MMSE: Mini-Mental State Examination
MSPSS: Multidimensional Scale of Perceived Social Support
PTG: Post-traumatic Growth
PTGI: Post-traumatic Growth Inventory
saBIC: Sample-size Adjusted Bayesian Information Criterion
STROBE: Strengthening the Reporting of Observational Studies in Epidemiology
WHO: World Health Organization
